# “I used to be someone else”: postpartum identity and invisible labor among urban mothers in Chennai, an interpretative phenomenological study

**DOI:** 10.3389/fpsyg.2025.1687880

**Published:** 2025-12-18

**Authors:** J. Priyadharshini, R. K. Jaishree Karthiga

**Affiliations:** School of Social Sciences and Languages, Vellore Institute of Technology, Chennai, India

**Keywords:** emotional labor, gender norms and role strain, maternal mental health, motherhood and career, postpartum identity

## Abstract

This paper investigates the identity disruptions and emotional labor experienced by postpartum working and once-working mothers in Chennai, South India, through an interpretative phenomenological analysis (IPA) of five in-depth narrative interviews, selected from a broader pool of 62 respondents in the screening test. While postpartum distress is typically conceptualized within a framework of clinical or psychological diagnosis, this research situates maternal suffering in socio cultural and gendered ways that shape women’s lived experiences. The five participants included women 25–35 years of age, who provided detailed and raw accounts that illustrated how early motherhood ruptured their self concept, affected their professional aspirations, and resulting emotional incongruence, even when there appeared to be family supports or accommodating workplaces in place. Across the narratives, themes of guilt, role overload, emotional invisibility, and internalized ideals of “perfect motherhood” emerged as dominant. Although some tried to cope by engaging in flexible work or family arrangements, most described ongoing frustrations in managing unequal domestic responsibilities and being displaced from their sense of self. The findings demonstrated how early motherhood when placed in unequal domestic and institutional systems can produce long-lasting identity loss and psychosocial strain. The study urges the need for a culturally relevant reframing of maternal mental health, moving beyond biomedical perspective toward structural awareness and subsequent policy reform. The findings highlight the necessity for reforming policy that not only addresses structural inequities in care giving but to promote maternal mental health as an essential component of public health strategy that aligns with Sustainable Development Goal 3.4, which seeks to improve mental well-being, and supports the World Health Organization’s emphasis on integrating perinatal mental health into maternal and child health services.

## 1 Introduction

Motherhood is not just a biological or personal phenomenon, but a deeply socio-cultural and political shift that transforms a woman’s identity, body, and time. Rich (1976) made a powerful distinction between the experience of motherhood and the institution of motherhood, arguing that societal scripts often override women’s lived experiences. This framing is particularly pertinent in the postpartum period, when women are expected to exhibit care, patience, and domestic competence, despite experiencing severe emotional or physical distress.

Motherhood alters women’s aspirations, constraining future goals and ambitions beyond the emotional and psychological shifts. Due to the overwhelming demands of care giving, many women experience a major shift in their life trajectories. Also, the transition into motherhood has significant economic consequences. Mothers in postpartum often face career interruptions and fewer opportunities for progression when they attempt to resume work. This causes diminished confidence along the burden of managing dual roles.

Mothers who intended to pursue education give up their aspirations due to lack of childcare support, rigid institutional systems and cultural expectations that prioritize family responsibilities over individual woman’s personal growth. These socio-economic pressure together highlights that motherhood is not a mere psychological transition but a structural turning point in a woman’s life which reshapes her autonomy.

The transition to motherhood often causes significant psychological, social, and identity changes, as women move from an independent sense of self to one that is influenced by the demands of caring for others and social norms (Laney et al., 2015). According to Rubin’s (1984) early theory of maternal identity, this transition involves disengagement from the former self and slowly reconstructing an identity around the child. Adding to this, identity theory offers a useful framework to explore the multiple competing pressures that postpartum women experience. Stryker and Burke’s (2000) framework, emphasizes that individuals hold multiple role identities such as “mother, professional, or daughter-in-law,” arranged hierarchically depending on context, salience, and social validation (Stryker, 1980). When one role dominates disproportionately, often due to social pressure, it can lead to identity conflict and distress (Thoits, 1991). Erikson’s (1950) theory of psychosocial development suggests that identity confusion, which is associated with adolescence, may reappear during life transitions like motherhood, when individuals question, “Who am I now?” (Marcia, 1966).

Hays (1996) elaborates on the dominant cultural script of “intensive mothering,” that is mothers are expected to be selfless, emotionally sensitive, and completely committed to their children. Although this ideal cannot be achieved often, it is frequently internalized, which results in identity erasure, burnout, and guilt. In this study, participants reflected this paradox. While trying to return to work or reclaim personal time, they reported experiencing feelings of guilt, judgment, or self-doubt that were emblematic of the emotional and cognitive labor demanded of today’s modern mother (Daminger, 2019).

Baraitser (2009) theorizes that the temporality of maternal care creates a suspended self, caught between social invisibility, routine, and repetition. This “stuckness” is represented in the stories where women described caregiving as an endless cyclical loop, “*I gave up space after space. And with it, I gave up on myself*” (Rekha). The psychic burden of this labor is often overlooked, both in the family and institutional contexts.

In particular, researchers worldwide recognize that maternal identity is shaped by sociocultural contexts. In their cross-cultural meta-synthesis, Dennis and Chung-Lee (2006) found a number of structures of support, stigma, and role expectations that mediate postpartum well-being, foregrounding how hegemonic, biomedical frameworks of postpartum depression obscure the depth and complexity of postpartum conditions. Likewise, both Miller (2005) and Apple (2006) also argue from a medical humanities approach, which recognizes not just pathology but also meaning-making, ambivalence, and emotional complexity.

These frameworks are especially important in urban India, where postpartum women experience a dual burden of traditional expectations and modern pressures. As participants in this study openly stated, maternal distress is not always visible or medicalized, but is often buried under emotional restraint, role conflict, and internalized guilt. Redefining maternal mental health in India requires acknowledging postpartum distress as a subjective and structural phenomenon rather than just a hormonal or psychological imbalance.

Bhaumik and Sahu (2025) illustrate how employed Bengali mothers engage in negotiating their emotional health with the expectations placed them to be caregivers. The findings of this study demonstrates how maternal subjectivity develops through the tension experienced by employed mothers as they try to balance their culturally designated roles as mothers and their personal fulfillment. In Chennai, these identity disruptions are amplified by traditional family expectations and modern professional commitments. Postpartum depression affects roughly 19–25% of women in Chennai, and it is made worse by family dynamics, a lack of social support, and urban isolation (Sita and Meenakshi, 2021). As more women join or return to the workforce, Chennai’s social structure still values intensive motherhood and domestic work, creating potential conflict with privately and publicly held gender expectations (Janiso et al., 2021). In such contexts, postpartum distress is usually diagnosed as depression or anxiety, which tends to take on a medicalized meaning, whereas socio cultural aspects such as the gendered division of labor, emotional invisibility, and generational ideas of the “perfect mother” have not been explored in depth (O’Reilly, 2016).

Although there is increasing recognition of the importance of maternal mental health across the world, India continues to have significant policy and implementation gaps in this area. The National Mental Health Programme (NMHP) and Reproductive, Maternal, Neonatal, Child and Adolescent Health (RMNCH+A) strategies on mental health focus primarily on maternal mortality from physical health standpoint, with limited reference to psychosocial or emotional well-being during pregnancy and postpartum. There is no national requirement for the public health system to screen routinely for postpartum depression or mental distress during antenatal or postnatal check-ups.

All indicators on maternal mental health are excluded from even one of India’s largest sources of health data, the National Family Health Survey (NFHS-5). The lack of critical data on postpartum distress, depression or anxiety at the population level (International Institute for Population Sciences and ICF, 2021) is unprecedented. Maternal mental health is not singled out and addressed at a policy level, especially considering the recommendations made by the World Health Organization (WHO) for mental health to be integrated into primary maternal health care (World Health Organization, 2022; United Nations, 2015). As cultural stigma persists, there is also a limited supply of trained mental health providers. Moreover, primary health care facilities in India carry a heavy burden of work. This has rendered postpartum psychological distress invisible for many women and girls, particularly in urban, lower- and middle-income contexts. Many women are left to suffer, without seeking help or even understanding their emotional needs, often dismissed as a “normal” aspect of motherhood and parental care.

This research investigates the lived experiences of postpartum women who were either employed or had been employed prior to childbirth. The sample consisted of 62 participants and the research drew five women aged 22–35 who provided shared narratives describing how early motherhood interrupted their self-identities, derailed their aspirations for work, and created a significant amount of emotional labor that they must engage. The research employs an interpretative phenomenological analysis (IPA) perspective to examine how these women navigate conflicting identities amidst issues of structural inequality, emotional invisibility, and judgment from others. This research engages maternal subjectivity, identity theory and feminist perspectives of normative motherhood to advocate reframing postpartum distress not just as a clinical issue, but rather a sociocultural crisis that requires transformative action at systemic and policy levels.

## 2 Method

### 2.1 Research design

This research used interpretative phenomenological analysis (IPA) to understand postpartum women’s experience in Chennai of identity transformation, maternal guilt, and emotional strain. IPA is specifically appropriate for understanding participants’ meaning-making processes during transitions in their lives and includes a dual-layered hermeneutic approach meaning that the researcher is able to interpret participants’ accounts of their experiences and the deeper psychological meanings contained within participants narratives (Smith et al., 2009). A purposive sampling strategy was used to identify mothers who had undergone significant postpartum emotional, psychological or identity related challenges. As IPA is primarily focused on the individual (idiographic) rather than the group, the study utilized a small sample of people.

### 2.2 Participants

Five postpartum mothers living in Chennai, Tamil Nadu, were purposively sampled through maximum variation sampling to represent a diversity of socio-economic status, employment history, and family structure. All five participants were either currently employed or had engaged in paid work prior to giving birth, which allowed the study to investigate how the postpartum maternal identity influenced the disruption or continuation of paid work. The five participants were finalized from the screening questionnaire spread through a variety of sources including personal networks, referrals from friends in the community and snowball sampling, allowing access to women willing to share detailed, intimate narratives. The participants reflected differences across income levels, professional fields, housing status and family structure.

All the participants had previous work experience. However, their employment histories varied based on the duration, the type of work and the ability to go back to work after childbirth, providing rich analytic diversity among the group.

None of the participants in this study were diagnosed with postpartum depression, nor were they undergoing any psychological or psychiatric treatment at the time of the interview. Participation was based on their own report of having difficulty with emotions and identity rather than clinical criteria.

#### 2.2.1 The inclusion criteria

1. Women aged 22 to 35 years.
2. Mother of at least one child under the age of three.
3. Willingness to participate in an in-depth interview of 60 to 90 min.
4. Competence in Tamil and/or English to communicate effectively.

This participant profile was intended to highlight the lived experiences of early motherhood in working or former working women, specifically the emotional and identity-related changes they experienced within the context of South India’s cultural and domestic spaces. Overall, the final sample consisted of five participants with moderate variation of women with different work trajectories post-childbirth (returned to work, not allowed to return, discontinued/career break), family types (nuclear, joint), and professional backgrounds (education, corporate, research, aviation). By including participants with varying employment statuses, family types, and professional backgrounds, the study captured the diversity of postpartum identity dislocations and caregiving circumstances while adhering to IPA’s idiographic approach to understanding individuals and how they make meaning of their experiences. To enhance transparency and facilitate greater understanding of the socio-economic variation, it was detailed in Table 1. Characteristics of the participants is provided just after this subsection.

**Table 1:** Participant characteristics table.

Participant	Education	Employment (pre-childbirth)	Employment duration	Religion	Return to work (post-childbirth)	Income range	Housing/Assets	First/Second child	Family structure	Spousal support	Extended family involvement
Chandra	B. A., B. Ed	Teacher	1 year	Christian	Not allowed	Low	Rented	First	Joint	Minimal	Daily
Rekha	Engineering	Chief Operational Officer at a private company	4 years	Hindu	Not returned	Middle	Rented	First	Nuclear	Good	Occasional
Pooja	M. A.	Cabin Crew	2 years	Hindu	Not allowed	Upper-Middle	Joint family assets	First	Joint	Good	Financial
Sree	M. A., M. Phil, NET-JRF	Assistant Professor	3 years	Hindu	Returned	Middle	Own house	First	Joint	Minimal	Daily
Aishwarya	M. Sc., B. Ed.	Lecturer	2 years	Muslim	Not allowed	Upper-middle	Own house	Second	Joint	Very minimal	Daily

### 2.3 Data collection

Data for the study were collected through a two-step process. In the first step, a screening questionnaire was sent to postpartum women living in Chennai, Tamil Nadu, using Google Forms. The questionnaire was sent to personal networks, community WhatsApp groups, and referrals in order to reach as many women as possible. The screening questionnaire contained demographic information, employment status before and after birth, and self-reported emotional or identity challenges faced in the postpartum period. The identification of socio-economic diversity, work history and family structure were identified through the screening questionnaire before selecting the participants for the interview. The following questions were asked:

*Section 1: Background information*

1. Your name
2. Age
3. Location (city/town/village)
4. Number of children
5. Age of youngest child
6. Type of family
  - Nuclear
  - Joint
  - Extended
  - Other (please specify)

*Section 2: Work history*

1. Were you employed before childbirth?
2. Are you currently working?
  - Yes, full-time
  - Yes, part-time
  - No, I’m on a break
  - No, I left work permanently
3. If you left your job or took a break, what were your reasons? *(open-ended)*

*Section 3: Experience indicators*

1. On a scale of 1–5, how much has motherhood changed your sense of identity?
2. Have you experienced any of the following after childbirth? *(check all that apply)*
  - Guilt or shame related to work or family
  - Pressure to be the “perfect mother”
  - Conflict between personal goals and parenting
  - Feeling “lost” or unsure about identity
  - Loss of self-worth or professional confidence
  - None of the above
3. Would you describe your postpartum journey as emotionally challenging, even in small ways?
  - Yes
  - Somewhat
  - No

*Section 4: Participation interest*

1. Would you be willing to participate in a one-on-one confidential interview (60–90 min, online or in-person)?
2. If yes, please share your email address or phone number to contact you.
3. Would you prefer to remain anonymous in the final published study?
  - Yes
  - No

The screening questionnaire shared via Google Forms, sent out with personal and professional networks in Chennai, only generated 62 responses. Out of the responses received, nine mothers met the inclusion criteria (postpartum within 3 years, aged between 22 and 35, currently working or worked before child-birth). Of the nine participants, five chose to share their full postpartum narratives as part of the study.

For phase two, the five selected participants, took part in in-depth semi-structured interviews. The semi-structured interviews lasted between 60–90 min, and they were scheduled either online or in-person dependent on participants’ preferences and availability, during which ensured a quiet and private space for the session. The interview examined six distinct domains:

1. Identity pre- and post-motherhood (gaining insight into changes in self-identity).
2. Work and self-identity (gaining insight into the disruption of professional identity).
3. Gender norms and expectations (gaining insight into societal and familial expectations).
4. Emotion and conflict (gaining insight into guilt, pride, ambivalence and tension).
5. Support and isolation (gaining insight into both emotional and infrastructural support).
6. Redefining success (gaining insight into evolving measures of self-worth).

Prior to starting the interviews, participants read an information sheet that explained the research, the procedures, the voluntary nature of participation, the measures to protect confidentiality, and that they can withdraw from the study at any time before 1 month after the interview, at which point their data would be discarded. The participants provided informed consent by signing a digital consent form. The participants were informed of the confidentiality protocol and details about the confidentiality limits. With consent, interviews were audio-recorded and transcribed verbatim using a password protected transcription tool. The recorded conversations were a mix of Tamil and English, which the interviewer notated and then transcribed and translated for analysis. Names of people, names of places, and any other similar potentially identifying information were coded out during transcript transcription to protect the confidentiality of the participants. The research design was such to provide ethical integrity while maintaining some richness of the narratives for analysis.

The interviews focused on several themes, including maternal guilt, emotional labor, loss of identity, professional loss, and societal expectations. This layered process ensured both breadth (via the screening survey) and depth (via narrative interviews) in understanding the lived experiences of postpartum identity disruption. After each interview, the participants were provided time to ask follow-up questions, and to add any other information they wanted to share.

### 2.4 Data analysis

Drawing on the phenomenological emphasis upon embodied experience and embodied perception espoused by Merleau-Ponty, the data was analyzed through the six-step method of interpretative phenomenological analysis (IPA) as described by Smith et al. (2009). Each transcript was read multiple times to allow for appropriate immersion, along with initial noting containing descriptive, linguistic, and conceptual comments. Emergent themes were developed for each case to grasp the essence of the participants’ lived experiences. The themes were grouped into superordinate categories to allow patterns across cases to be identified while still being mindful of individual nuance. Enduring reflexivity was maintained throughout the research process, complemented by analytic memos documenting the researcher’s shifting interpretations and positionality. The final thematic structure connotes convergence and divergence in postpartum women’s understanding of identity, caregiving, and selfhood.

## 3 Phenomenological approach and interpretative phenomenological analysis

Phenomenology is a qualitative research approach that specifically examines how individuals understand and make meaning of certain phenomena in depth. Phenomenology, based in the philosophical traditions of Edmund Husserl and Maurice Merleau-Ponty, uses lived experience as the basis for understanding, rather than objective descriptions, or theoretical assumptions (Husserl, 2001; Merleau-Ponty, 1962). The postpartum period is often characterized by drastic changes in emotional stability, psychological adjustment, and identity shifts, and is a prime candidate for a phenomenological inquiry, especially as experiences in the postpartum period are social and embodied in nature.

The body, Merleau-Ponty (1962) asserted, is not just something in the world, but our means of access and experience in the world. This embodied subjectivity holds special relevance in the case of motherhood as changes in physicality, social roles, and identity are wholly interdependent. In the case of maternal feminist phenomenology, converging on women will allow the silent metamorphosis of women’s experiences be discovered, something that is suppressed due to the normative narratives of joyous motherhood (Kukla, 2005).

Postpartum experiences involve significant emotional labor that often goes unexamined for a variety of reasons, including culturally driven taboos and internalized expectations. IPA is particularly well-equipped to challenge these silences, not just the information that mothers convey, but also the information, emotion, and integrity that mothers exercise by withholding. Benuyenah and Tran (2020) conducted an IPA study on the experiences of single mothers from Vietnam. In interviews, participants expressed significant psychological pressure to conform to external expectations and societal judgment through episodes of disjointed storytelling and refraining from sharing their feelings regarding the experience of motherhood in conjunction with being a single mother. While it is important to listen carefully to what participants means about their experience, IPA is designed to capture the emotional nuances embedded in mothers’ tone, silences, and metaphors.

Sleep deprivation is often a universal postpartum experience, but is usually trivialized in terms of its psychological impacts. Chwalko (2019) conducted an IPA study on how first-time mothers in the UK understood their sleep loss. While deprivation was thought of in relation to sleep, mothers described sleep loss as a physical and psychological sleep, presenting itself as a disruption in identity. Mothers described an experience of feeling “disassembled” post-baby and noted the challenges in making sense of who they were pre-baby and having to be a mother. While IPA is not a form of transformational analysis, it lends itself to some consideration of how attributes such as sleep deprivation interact with self-worth, relational position, and maternal competence.

A study by Newson et al. (2017) in the UK using IPA showed that women often cycled between feeling proud of their body’s ability to function and feelings of shame regarding its altered appearance. Participants reported feelings of tension to “bounce back” and still expressed amazement at their body’s ability to support life. The value of IPA as a method is in capturing these contradictions, giving researchers the opportunity to contemplate how women are negotiating self-perception through a cultural lens and a personal lens.

This study employed interpretative phenomenological analysis (IPA) to explore how working mothers or formerly working mothers in Chennai make sense of their postpartum experiences and new identities. IPA is particularly useful for understanding how individuals make sense of their experiences during significant life transitions informed by personal, social and cultural contexts (Smith et al., 2009). This method allows for an idiographic and in-depth analysis into meaning-making of each participant rather than the empirical search for universal truths.

IPA proceeds with a double hermeneutic, where the researcher is coming to terms with how the participant is coming to terms with their lived experience (Smith and Osborn, 2008). This is of particular importance for my study because the participants expressed cultural nuances and multifaceted meanings related to guilt, doubt, and resilience. The researcher is aware of her own positionality as a South Indian woman who has similar cultural reference points. The researcher also merely tried to bracket her own biases by recollecting her reflections of bias in memos after each interview (Finlay, 2002).

In addition, IPA allows for the investigation of cultural expectations and internalized norms in the participants’ stories. For instance, although several participants had difficulty articulating feelings such as shame, isolation, and loss of self, those lived experiences came through clearly in metaphor, anecdote, and silence. This is consistent with van Manen’s (2014) proposition that phenomenological research exposes “the taken-for-granted meanings in lived experience.” In societies where maternal mental health is remaining taboo, IPA provided a valuable opportunity to bring to life unspoken or socially censored aspects of postpartum experience.

This approach is frequently employed in maternal health studies worldwide, including studies of post-natal depression (Cirban et al. 2025), ambivalent motherhood (Charmaz, 2014), and negotiating identity (Laney et al., 2015). However, IPA has been largely untapped in Indian contexts, particularly in relation to the urban educated woman who is traversing motherhood amidst the loss of her professional ambitions.

Thus, employing IPA not only affords the most methodologically sound approach but also ensures that the voices for Chennai’s mothers are at the center. They speak to larger tensions between tradition and modernity, family duty and individual agency, love and loss in the process of becoming a mother.

## 4 Thematic analysis

This section describes the main themes that emerged from the interpretative phenomenological analysis of five postpartum narratives. Each theme reflects the participants’ lived experiences of identity disruption, emotional labor, and cultural expectations in the early months and years of motherhood. Although every narrative is distinctive, there were collective themes of guilt, invisibility, and strain across all cases. Each theme is supported by direct quotations to maintain authenticity of voice and show the emotional texture of each experience.

The themes are interwoven and mutually reinforcing rather than functioning as isolated categories; for example, disruption in identity intensify emotional invisibility, which in turn deepens guilt and thus aggravate career sacrifice. The inter-relationships between these themes are pointed out throughout this section to illustrate the overarching phenomenon of postpartum identity erosion.

### 4.1 Disruption of identity and selfhood

Many postpartum women in this study felt their previous identities in professions, social roles or aspirational roles had been totally swallowed up by the maternal identity they now wore. Many of the participants described a jarring displacement: being a “mother” trumped every other identity, and as an identity, there was no other space for them, or the opportunity to negotiate the “self.”

This foundational disruption underlies several later themes such as career sacrifice (4.2) and emotional invisibility and isolation (4.3), suggesting that identity loss is not a singular experience but a core condition shaping every subsequent struggle. When they were asked to explain their identities before and after motherhood, the participants concluded:

“*I had goals. Now, I don't even know who I am. I sometimes wonder who I am now, beyond being a mom. I gave up space after space. And with it, I gave up on myself. I used to be respected at work. Now I doubt myself. I look in the mirror and don’t see someone successful anymore. I wonder if I can ever go back to work again. Will I ever be good at my job again?*”—Rekha

“*I don’t care for myself anymore. I put my daughter first.*”—Sree

“*This is life, I’ve accepted it. Even if I want to change something, nothing moves. I’m not sad. But I’m not me anymore. Some days I look in the mirror and don’t recognize the person. Not just physically, even mentally I’m not myself.*”—Chandra

“*I don’t feel like myself. I don’t laugh the way I used to.*”—Pooja

“*I used to be the first to speak up. Now I just fade in the background. I feel like I lost who I was. All I do now is feed, clean, and worry.*”—Aishwarya

There is a continuing feeling of self-loss, a dislocation in time between the woman who existed before, and the woman you are now. The participants described a split, a confident, ambitious woman who is now reduced to a disembodied, silenced, and broken maternal identity. These identity ruptures echo in later themes showing that emotional, relational and cultural struggles are not separate issues but essence of the same core identity distruption. This reflects Merleau-Ponty’s concept of lived body, that identity is embodied, and the bodily changes that occur in motherhood destabilize women’s sense of being, but also Baraitser’s notion of maternal suspension, where time, ambition, and selfhood are indefinitely suspended.

### 4.2 Career sacrifice, silenced, and suppressed dreams

As identity rupture is the nucleus to all the narratives, the next significant expression of this disruption emerges through career sacrifice and the erosion of professional identity. For several women, the collapse of selfhood lead to slow or sudden retreat from professional identity ranging from stalled ambitions, missed opportunities to full withdrawal.

“*I wanted to do B.Ed. first. But I couldn’t even convey it at home.*”—Pooja

“*Everyone says ‘we’ll help,’ but I end up doing everything. I wanted to switch jobs but felt stuck in a caregiving loop. I’m a JRF candidate and I wished to do a full-time Ph.D. I sacrificed my dream and choosing a workplace which runs for half a day is again for the sake of my child.*”—Sree

“*I kept saying ‘soon, after he goes to school, I can work,’ but that ‘soon’ never comes.*”—Aishwarya

“*My career took a hit. I didn’t apply for the promotions I deserved. I once dreamed of being powerful, then, one project seemed too much. I had to quit my job. My plans just disappeared.*”—Rekha

“*I always feared, ‘what if I’m failing both my child and my job?’*”—Rekha

“*I was preparing for government exams, I couldn’t even study for two months after delivery.*”—Chandra

Professional identity is consistently depicted as sacrificed. The maternal obligation is viewed as being mutually exclusive of the ambition. While supports may exist, expectations and guilt lead women into sacrificing elements or disengaging completely. These experiences reinforce what Gill and Scharff (2011) call postfeminist failure narratives; women are told they can “have it all,” while they do not receive the necessary structural and emotional support to hold down both caregiving and career. In this context, Berlant’s (2011) concept of “cruel optimism” is particularly resonant: the ideal of effectively navigating motherhood and ambition becomes emotionally damaging in the absence of social conditions that allow one to live the ideal. The experiences of participants are also consistent with Júlia et al. (2024) findings, who point to systemic barriers and cultural expectations that contribute to a “motherhood penalty” which prevents several career-related opportunities. In various Asian contexts, Bourcet Nguyen (2024) discusses how, the expectation of maternal obligation is often seen as incompatible with leadership or upward trajectory in careers, exacerbating identity conflict and professional wear and tear over time.

#### 4.2.1 Professional disintegration and identity collapse

Rekha’s journey from COO to full withdrawal is not simply a career loss, it’s about the dissolution of a once-secure identity. This subtheme examined how caregiving demands erode professional roles from the center of self-definition, resulting in existential disorientation.

“*I was the COO of a company, managing multiple departments. I slowly gave up my role in office, from COO to managing one project to nothing. I didn’t even realize when I stopped being that person. Now, I’m home all day.*”—Rekha

This shows identity foreclosure where external roles are resigned without internal deliberation. As Baraitser (2009) describes it, this is a kind of maternal suspension that goes on; Giddens (1991) described a rupture of self-narrative when continuity of self is interrupted.

### 4.3 Emotional invisibility and isolation

This emotional withdrawal grows out of identity loss and curtailed aspirations, deepening the sense of erasure that these women already experience. Participants described a sense of emotional distancing from the world around them, shrinking social circles, and loss of relational energy. Hochschild (1983) described a scenario where postpartum women experience what she termed “emotional overload” whereby empathy and care is always given but not returned.

“*I was once bubbly. Now I avoid even calling friends. My circle has shrunk. I feel I’ve become invisible.*”—Pooja

“*I don’t want to talk to anyone. I’m tired of explaining.*”—Aishwarya

“*Even when I’m sick, I feel guilty resting. Who else will do the work? I was just giving and giving, but nobody noticed.*”—Sree

“*After delivery I was left alone to deal with the child. When he cried, I wanted to run away from home.*”—Rekha

These narratives emphasize emotional exhaustion, disconnect, and a lack of empathetic listening in their surroundings. This is consistent with the notion of “affective invisibility” (Rose, 2019) and how caring labor, and particularly emotional labor in this instance, becomes imperceptible.

### 4.4 Guilt, ambivalence, and emotional overload

The emotional invisibility described by the participants flows into an intense psychological landscape, i.e., guilt, ambivalence, and emotional overload, revealing how identity and relationship disruptions lead to internalized self-blame. All participants experienced guilt for not being perfect, either at home or at work. Many mothers expressed deep ambivalence, yet lacked the emotional or cultural space to process it. As Almond (2011) argues, maternal ambivalence remains a taboo, despite being a universal emotional reality.

“*If my baby fell or cried, they’d say I was distracted. That guilt never leaves you.*”—Pooja

“*Sometimes I shout at my child, and immediately regret it. Then I sit and cry after he sleeps.*”—Aishwarya

“*Even a phone call from an employer made me feel torn. I kept thinking, ‘should I be here or there?’ I felt guilty for not being present enough, both at work and home.*”—Aishwarya

“*When I rejoined work after maternity leave, I was scared they’d think I couldn’t handle work anymore. I kept pushing myself to prove I’m still capable.*”—Rekha

“*I felt I was not doing justice to either. I had to leave work when my son’s speech delay worsened. The guilt of leaving my child was stronger than ambition.*”—Rekha

“*I don’t want my parents to know I’m struggling.*”—(Pooja, Aishwarya)

“*Not one person said I’m not a good mother but everything they do makes me feel it. Earlier women went through domestic violence or verbal abuse, now it's even worse as people outside think I’m happy, I have a family to support or take care of my child and I’m successful as I work but the reality inside the four walls is horrible.*”—Sree

“*If something went wrong with the child, food habits or any act, I’m blamed. When my child does any good act, the credit goes to my husband. No one said I was a bad mother, but their reactions or indirect comments do.*”—Sree

“*I constantly think, ‘Why is only my identity erased in child care?’*”—Chandra

Participants described an ongoing emotional holding pattern, not in the form of a mental illness, but as an ongoing grief for the parts of themselves that were lost, a phenomenon Siebold (2025) describes as postpartum identity loss, in contrast with clinical depression. There is no vocabulary for being “not okay’ in motherhood without shame of judgment, as Llana (2024) notes in her critique of the cultural ideals that silences the complexity of motherhood. The guilt they were carrying was not only emotional, it was structural, constructed by the myth of the perfect mother, the expectations to be everywhere all at once, and a lack of space to voice ambivalence. The “Perfect Mother Myth,” highlighted by Jeanna Boase (2025), sustains emotional suppression and erases the transitional identity work of matrescence.

### 4.5 Gendered labor and unequal parenting

Much of the guilt expressed by the participants is rooted in structural inequalities within caregiving. Unequal domestic labor, the cognitive load, and patriarchal norms become the mechanisms that intensify emotional overload and identity strain. The labor of motherhood was not equally shared. The participants across the narratives spoke about unequal domestic labor, and highlighted that despite the women taking on jobs, they are usually left to complete the domestic labor and childcare. Gendered labor division fuels burn out, which is damaging to the feeling of shared responsibility. The unequal division of both domestic responsibilities and parenting responsibilities continues to mirror the second-shift feminism perspective, described by Hochschild and Machung (2012). And while there is a physical labor division, the emotional labor and cognitive labor of organizing, planning, anticipating is all considered maternal. The emergence of mental load as a gendered burden goes largely unseen and creates emotional distress.

“*When I said ‘I’m going to work,’ I was expected to finish all the household chores, take care of my in-laws, bathe and feed my child, and prepare food before leaving and then complete the remaining chores after returning home, just because I chose to work. I’m a female, a daughter-in-law, a wife, a mother and it is my duty.*”—Aishwarya

“*My husband thinks helping once is enough. But parenting is daily and it is not a help!*”—Aishwarya

“*I do everything. My husband will help only if I ask 3 times.*”—Aishwarya

“*Even if my husband is at work from home, it is my duty to do all the chores and make everything ready for my child before leaving for work. My in-laws do not allow my husband to help me in childcare just because he is a male.*”—Sree

“*Even today, my son’s needs are seen as ‘my’ duty. My husband helps, but the mental load is mine. I carry the list. He’ll do it only if I say. Why cannot he think of it himself?*”—Rekha

“*He’s praised for doing the bare minimum.*”—Pooja

These accounts reveal that patriarchal norms still allocate parenting labor to women, regardless of whether they are employed. The quotes illustrate the concept of the “mental load” (Daminger, 2019), a specific cognitive load that is excessively borne by mothers. The second shift is not just about the physical chores, but also planning, worrying, and preparing.

### 4.6 Cultural pressure, moral policing, and maternal judgment

The wider cultural system is behind all the above themes that defines and evaluates the maternal behavior. Cultural pressure and intergenerational judgment are the structural forces that not only legitimize unequal labor but intensify guilt and erase one’s identity.

Maternal success is narrowly defined. Traditional gender scripts and intergenerational beliefs impacted their self-image. Participants mentioned the strong presence of cultural expectations even among families who live in urban or progressive space and the family, in this case, included in-laws too. Participants described both harsh observation and criticism, as well. They also talked about how intergenerational expectations, particularly from family members and in-laws and culturally defined scripts of what “good motherhood” looks like, outlined “good motherhood” in rigid terms.

“*If I raise my voice, they say I’m a bad mother. They blame me even if my child pulls anything off the shelf. I’m asked to be in 24/7 surveillance. They make me feel like I’m always doing something wrong.*”—Pooja

“*My mother says a good mom wakes early, toilet trains, and never shouts.*”—Pooja

“*My mother-in-law would say, ‘You should’ve done this or that.’ No one appreciated what I was doing. They criticize me for everything: how I feed, how I hold him, what I cook for him.*”—Aishwarya

“*I’m constantly asked, ‘Why can't you be like other mothers?’. There are constant comparisons with my sister-in-law. From changing diapers or giving medicines to my child to my decision to go to work after delivery. They expect me to be a home-maker like their daughter. They made me feel like a failure for not being like her. They criticize me for everything: how I feed, how I hold him, what I cook for him.*”—Aishwarya

“*Speech delay, maybe it was because I gave too much screen time to focus on work peacefully. I felt like I compromised his health.*”—Rekha

“*No one openly says I’m failing, but there’s always an unspoken judgment.*”—Rekha

“*Although no one said I was a bad mother, their reactions or indirect comments do. They said the child didn’t eat because I went to work.*”—Sree

These quotes demonstrate how intergenerational and cultural beliefs transform postpartum experiences into sites that are morally evaluated and regulated. Guilt becomes a prominent emotion and is not merely internalized, but rather, is extensively socially constructed and socially regulated, often intergenerationally with respect to then social expectations and patriarchal surveillance. In this sense, it echoes Berlant’s “cruel optimism,” where the fantastical and unattainable idea of perfect motherhood produces emotional self-harm, and Davis’ idea of “the feminine mystique,” where the myth that satisfaction comes solely from domesticity and sacrifice.

## 5 Discussion

The results of this research indicate how postpartum identity disruption is not only a psychological shift but rather a fundamentally social and culturally bound experience. This section interprets the themes through a feminist, sociological, and phenomenological lens to understand how caregiving, cultural norms, and emotional labor are components of maternal subjectivity.

“*Why is only my identity erased in child care?*”—Chandra. This rhetorical question encapsulates the essence of what all these narratives reveal, that motherhood under the current social understanding is a location of identity erasure unless conceptualized differently with support, equity, and understanding.

The findings provide a critical lens into the emotionally complex, socially inextricable and psychologically disorienting experiences of postpartum women, especially women who had previously held or were seeking professional identities. Although participants are from different family arrangements and with different professional identities, the narratives emerge around the internalization and distortions of unequal norms of domestic expectations, a disrupted identity, and the overwhelming nature of maternal perfectionism.

Each theme contributes to the study’s primary objectives. Themes 1 and 2, selfhood, and professional identity are primarily about how the demands of caregiving impact one’s sense of self determination; Themes 3 and 6, emotional invisibility and cultural stigma are about social scripts that dictate maternal behaviors; and finally, the guilt and ambivalence of Theme 4 reinforces the emotional burdens of mothering in an unequal world.

In all five narratives, the erosion of identity is palpable. Participants such as Rekha (“*I had goals. Now, I do not even know who I am.*”) and Chandra (“*Some days I look in the mirror and do not recognize the person. Not just physically, even mentally I’m not myself.*”) highlight what Arendell (2000) called the “dislocation of self” in the transition to intensive mothering. These women are not merely experiencing role transition; they are mourning a lost self. Baraitser’s (2009) notion of motherhood as a site of ethical interruption that divides the self, in favor of an other (the child) but little cultural recognition of that loss, rings true. What makes this experience more painful is the invisibility of the rupture. In the words of Pooja, “*I feel I’ve become invisible,*” echoed by Sree’s remark, “*I was just giving and giving, but nobody noticed.*”

Participants such as Aishwarya and Rekha powerfully demonstrate the contradictions of postfeminism, especially the false promise that women today can “have it all.” Aishwarya states, “*When I said ‘I’m going to work’, I was expected to finish all the chores. just because I chose to work.*” Aishwarya’s words illustrate how the neoliberal ideal of choice is lackluster; it is choice without structural context or cultural change. This is consistent with Gill and Scharff’s (2011) criticism of postfeminism, see it as covering up structural gender inequality by framing it as the fault of the individual. Rekha’s spiral from a respected COO to fearing she will never return to work is an illustration of what Rottenberg (2018) calls “the exhausted woman,” who we celebrate as empowered, but who is burdened by implausible workplace and home expectations.

Sree states, “*No one said I was a bad mother, but everything they do makes me feel it.*” Similarly, Rekha experiences internalized guilt for her child’s speech delay and says, “*I felt like I compromised his health.*” These sentiments draw on the ubiquitous cultural script of linking a child’s wellbeing back to maternal “performance”. Hays (1996) and Douglas and Michaels (2004) describe the “ideology of intensive mothering,” which holds mothers to impossible standards, and thus, guilt and shame become not incidental character traits but structural features of motherhood. This is exacerbated with intergenerational expectations, as evidenced by Pooja’s comment, “*As my mom says, a good mom wakes early, toilet trains, never yells.*” These findings echo Bhaumik and Sahu’s (2021) argument that maternal guilt, emotional overload, and the erosion of selfhood are not individual psychological failures but socially produced outcomes shaped by cultural ideals of intensive mothering in India.

While all of the participants in this study ostensibly had male partners or family support, they experienced a staggering asymmetry of caregiving labor. The mental load is both invalidating and exhausting, not the least of which is that the workload often casts doubt upon a woman’s worth in a realm beyond caregiving. As Rekha notes, “*The longer I stayed home, the more difficult it was to believe I was worth something outside of motherhood.*” Further, Pooja’s comment, “*He gets praised for doing the bare minimum,*” and Rekha’s frustration, “*He’ll only do it if I suggest it. Why cannot he think of it himself?*” make clear that, as Daminger (2019) identifies, it is the cognitive dimension of gendered labor that receives very little acknowledgement; planning, remembering, and emotional worrying about the caregiving needs of the household are things women disproportionately shoulder. The narratives simultaneously show, not just a disproportionate reallocation of physical labor in caregiving, but a deep erasure of cognitive agency.

Aishwarya’s comment, “*I get criticized for everything: how I feed, how I hold him, what I make for him,*” exemplifies motherhood as a public performance measured against intergenerational and patriarchal standards. Foucault’s (1977) concept of panopticism is apt in this situation. Mothers internalize other people’s gaze, even if there is no overt criticism, they alter their behaviors to avoid threats, even if it is for matters of daily life. These judgment processes operate on behaviors but also encroach upon emotional expression, what Pooja noted by saying, “*If I raise my voice, they say I’m a bad mother.*” The maternal self is bounded not by self-reflection, but in regards to the maternal self’s behaviors, compliance, comparison, and moral failure.

The women’s stories share in their broken aspirations, and we can see, from Sree’s delay of obtaining her PhD, to Rekha’s diminishing involvement as a leader, how aspirations were under the demands of care work and sustained caregiving expectations. Notably, none of these were “true choice,” they each arrived at these choices driven by guilt, peer pressure, and absence of material support; hope is met with suspension. For example, Aishwarya’s reflection, “*I kept saying ‘soon. I can work,’ but that ‘soon’ never comes.*”

Time itself becomes uncertain, echoing Baraitser’s (2009) concept of maternal time as repetitive, stuck, and unlike the progressive time of ambition and work. For Baraitser, maternal time is unstructured and without any predictability where emotional status of stasis becomes normal and normative. Here, the dissonance of time plays a significant role in loss of self; as participants struggle to show that there was a time in their past when they had hopes and plans for the future, and how the present and the uncertainty of it, enlisted and obliterated possibility. The future was postponed by both social policies and the emotional means to act liberalism, ambition and opportunity as a mother, and also a working one.

One side exposes the empowerment of feminisms social change: access to education, employment, and social participation and voice. The other side is proposed a cultural narrative that demands overarching availability, emotional dedication, and domestic willingness to comply. Thus these women are not simply exhausted; they are engaged in what Berlant (2011) referred to as “cruel optimism” where the very aspirations they are trying to materialize are in fact failing to do so, and become the source of their pain. The postpartum narratives of these mothers seek to illuminate how, for contemporary Indian mothers, and especially working and educated ones, the social landscape is fraught with contradictions.

The stories presented here interrogate dominant maternal success and mental health by centering emotional complexity, structural inequality, and cultural contradiction. They emphasize the need to reimagine motherhood, not as a space for silent sacrifice, but as a space for conflictual identity, ambition, and care. The first step toward creating more equitable and supportive maternal care is to understand these tensions.

## 6 Implications

The stories shared in this study provide important insights into the emotional, social and psychological costs of modern motherhood, especially for women formerly working in professions. The findings in this study have significant implications for healthcare systems, workplace organizations, gender discourse, and knowledge for future research. They demand a shift away from an understanding of postpartum distress as purely private or clinical phenomena, and to consider it as both a cultural and structural crisis.

### 6.1 Rethinking maternal mental health in healthcare settings

When the participants spoke of feelings like, “*I feel like I lost who I was,*” “*I do not even recognize myself,*” and, “*I’m not me anymore,*” indicate that postpartum distress is larger than we think the pathology of postpartum depression is defined in clinical models. This is likely more than just a psychiatric symptom, but rather a psychosocial crisis emerging from gendered work, emotional isolation and erasure of identity. This should trigger a larger, culturally situated understanding of maternal mental health in India, considering emotional, relational and sociocultural problems alongside the biological. Healthcare providers need to be trained to provide postpartum depression screening, but to also listen for signs of identity distress, guilt, and social invisibility. Maternal mental health should be situated in standard antenatal and postnatal care following WHO’s guidance.

### 6.2 Workplace reform beyond maternity leave

Postpartum identity disruption must be recognized as a professional obstacle rather than a personal shortcoming. Participants like Rekha and Sree revealed how ambition silently perishes when confronted with unfair domestic expectations. Their stories show that “maternity benefits” or “leave policies” are insufficient. Policies remain symbolic in the absence of cultural changes in gender roles and caregiving conventions. Workplaces must evolve from compliance to transformation by attempting: flexible re-entry programs, particularly for mothers returning after long gaps; mental health support groups at work for postpartum women; gendered policies that address the mental load and invisible labor.

### 6.3 Intergenerational education and cultural conversations matter

Mothers like Pooja and Aishwarya reflected on burdens they carried from cultural beliefs assigned to them from previous generations. This reaffirms the necessity of community discussion and intergenerational education in order to examine and question conventional notions of motherhood. Along with public health campaigns and maternal support networks, or prenatal education sessions, segments on deconstructing the myth of the perfect mother and responsible parenting could be included while promoting equal concern for individual parenting responsibilities in order to transform responsibility from maternal sacrifice into collective caregiving.

### 6.4 Including maternal voices in public discourse

These voices should not be hidden within the walls of academic journals. Their experiences of lost social circles, shame around displaying anger, guilt about their feeding decisions should be added to the public discourse. This study contributes to feminist knowledge making by bringing to the surface everyday maternal pain that is either normalized or silenced. It calls for: Media organizations to change the narrative of motherhood stories to reflect conflict and contradiction rather than idealism; Educational curriculums (specifically in education and gender studies) to include narratives about real life postpartum experiences that challenge theory; and, NGOs and maternal health advocates to have lived experiences at the forefront of program design and outreach.

## 7 Limitations

The study included five participants selected from nine eligible out of 62 screened. While the initial sample size is consistent with IPA’s focus on depth over breadth (Smith et al., 2009), and the intentionality behind purposive selection limits generalizability, this study was not intended to produce generalizable claims but rather thick, idiographic narratives of postpartum identity disruption.

All participants were cisgender, educated, Tamil-speaking women from urban or semi-urban populations in Tamil Nadu. Therefore, the cultural, linguistic, and socioeconomic diversity of perspectives is limited. Experiences of postpartum identity disruption vary immensely, for instance, within different community and class aspects of caste, across rural and urban divisions, and in forms of non-heteronormative family structures, which heretofore are not represented in this phase.

It is also important to clarify that the focus here is on mothers, as per the sample design, as mothers’ perceptions and experiences within the postpartum period were being investigated. Partners, in-laws, and employees often shape experiences of motherhood and postpartum identity through work place environments, dialog, and support, or surveillance. Future studies are invited to expand maternal narratives and triangulate with modes of co-parental, familial, or institutional participation or engagement, to capture shared and potentially conflicting narratives within care, judgment, and accountability.

Like all research studies, critical reflexivity and cultural bracketing were implemented, to support awareness of researcher positionality. Nonetheless, the goals of critical reflexivity may need to remain an active thinking process, as some common socio-cultural points of reference rendered themselves to enhance interpretation. By continuing to remain critically reflective, and transparent in conversions with further scholarship (Chang et al., 2016) both ontology and epistemology will be available. Overall, future considerations of the focus on participants will continue to inform our, as well as, intertextual scholarship on mothers’ experiences during the postpartum period.

## 8 Conclusion

The aim of this study was to consider the three objectives of: (1) how women with professional identities experience changes in selfhood after postpartum; (2) the emotional and cultural pressures that constitute maternal distress; and (3) addressing feminist scholarship through research that privileges lived experience. Reflected in five in-depth interviews in Tamil Nadu, the findings revealed that caregiving demands, gendered expectations, and culturally sanctioned ideals of maternal success disturb personal identity and emotional wellbeing.

The narratives reflected shared experiences, like the self that was immediately formerly was anchored to ambition, autonomy, and social connectedness, was displaced by caregiving demands, gendered labor, and moral expectations. Statements like, “*I feel like I lost who I was,*” (Aishwarya) or “*I was the COO, now I’m just home all day,*” (Rekha) illustrated dislocation from selfhood that arises not just from biologically needing to recover, but arising out of the emotional burden of being solely responsible for care (O’Reilly, 2016; Baraitser, 2009). Their experiences confirm Rich’s (1976) argument that motherhood is an experience and an institution—but it is an institution that often works against women’s self-definition and agency.

Recent feminist scholarship has critiqued biomedical models that reduce postpartum distress to hormonal changes and psychiatric disorders. Feminist scholarship advocates a reconceptualizing of postpartum mental distress as socially, emotionally, and structurally contained (O’Reilly, 2016; Daminger, 2019). Participants in this research described that their terrible emotional pain was compounded by domestic inequities, intergenerational judgment, and the difficulty balancing ambition with care; which is strikingly similar to Berlant’s (2011) notion of “cruel optimism,” where the very things women aspire to in ultimately cause them distress.

While the participants were victims in some sense, it is important to note they were not wholly passive victims. Their depictions showed ambivalence, resistance, and brief moments of clarity in the context of constraint. Some resisted the poisonous cultural demands while others mourned either dreams deferred or silencing. The women’s stories provide compelling feminist testimonials not just revealing what was taken from them but also what is still ongoing and being fought and articulated, in silence every day often invisibly.

This research adds to the fields of maternal studies, gender sociology, and feminist psychology because it demonstrates the narrative voice as a legitimate form of knowing and, as such, disrupts dominant notions of good mothering and exposes how women internalize structural inequality as individual deficiency. As Rich (1976) and Baraitser (2009) maintained, recognition is the first step toward change. This paper aims to make visible the emotional, spatial, and identity-based compromises women are burdened with in a world that sees maternal commitment and devotion as a form of self-erasure.

## 9 Scope for further research

This study provides profound insights into experience of urban, educated postpartum mothers in Tamil Nadu, while also paving the way for numerous avenues for future research. Any expansion of this study could deepen our understanding of the diversity and messy realities of maternal identity in India, as well as in global contexts.

### 9.1 Rural and semi-rural contexts

Future research could examine how postpartum identity disruption occurs in rural spaces as, for example, economic precarity, nested families, and restricted access to health care, would each likely produce maternal distress in a different manner.

### 9.2 Non-heteronormative families or single mothers

Experiences of single mothers, LGBTQ+ parents and non-heteronormative families, continues to remain unexplored in Indian maternal health research. Including these voices in the literature would expand feminist conceptualizations of care, stigma, and resilience.

### 9.3 Longitudinal studies

Longitudinal studies that address identity changes from birth, to short space of time after delivery to early childhood, could better document the trajectory of the continual process of maternal subjectivity from one change to the next, including where maternal subjectivity is detached in momentarily different futures. These studies could also document the re-entry into a previous professional/working space and how complex ambition is subsequently negotiated. A more interdisciplinary approach—one that draws on feminist value, phenomenological experiments like IPA, medical humanities and, social work could provide tremendous depth and breadth to maternal identity work.

### 9.4 Triangulated perspectives

Incorporating the multiplicity of partners, in-laws, employers, and healthcare providers would add greater substance to understand the postpartum ecology or dynamics of support, scrutiny, and shared responsibility.

### 9.5 Interdisciplinary approaches

A synthesis of feminist theory, IPA, medical humanities, and social work can create both scholarly and practice based knowledge. Creative collaborative research beyond disciplinary boundaries may also inform policy and program design which is more inclusive.

## Data Availability

The original contributions presented in the study are included in the article/Supplementary material, further inquiries can be directed to the corresponding author.
