# Supplementary material for: “I used to be someone else”: postpartum identity and invisible labor among urban mothers in Chennai, an interpretative phenomenological study
Source: Front Psychol. 2025 Dec 18;16:1687880. doi: 10.3389/fpsyg.2025.1687880 (PMC12756369; doi:10.3389/fpsyg.2025.1687880)
Supplement: Supplementary file 1 [file Supplementary_file_1.docx]

**Identity related statements:**

“I had goals. Now, I don't even know who I am.”

“I don’t care for myself anymore. I put my son or daughter first.”

“This is life, I’ve accepted it. Even if I want to change something, nothing moves. I’m not sad. But I’m not me anymore.”

“I don’t want my parents to know I’m struggling.”

“I was once bubbly. Now I avoid even calling friends. My circle has shrunk. I feel I’ve become invisible."

“They never directly called me a bad mother, but comments about food or screen time made me feel constantly judged.”

"I sometimes wonder who I am now, beyond being a mom."

"I don’t feel like myself. I don’t laugh the way I used to."

"I don’t want to talk to anyone. I’m tired of explaining."

"I used to be the first to speak up. Now I just fade in the background."

"At work and home, I became ‘mom’ before I could be ‘me’."

"I gave up space after space. And with it, I gave up on myself."

“I feel like I lost who I was. All I do now is feed, clean, and worry.”

**Participant 1**

“If I raise my voice, they say I’m a bad mother. They blame me even if my child pulls anything off the shelf. I’m asked to be in 24/7 surveillance. They make me feel like I’m always doing something wrong.”

"He’s praised for doing the bare minimum."  - Gender

“If my baby fell or cried, they’d say I was distracted. That guilt never leaves you.”

“My mother says a good mom wakes early, toilet trains, and never shouts.”

"I wanted to do B.Ed. first. But I couldn’t even convey it at home."

**Participant 2**

“My mother-in-law would say, ‘You should’ve done this or that.’ No one appreciated what I was doing.”

“ I’m constantly asked, ‘Why can't you be like other mothers?’. There are constant comparisons with my sister in law.  From changing diapers or giving medicines to my child to my decision to go to work after delivery. They expect me to be a home-maker like their daughter. They made me feel like a failure for not being like her. They criticize me for everything: how I feed, how I hold him, what I cook for him.”

“When I said ‘I’m going to work,’ I was expected to finish all the household chores, take care of my in-laws, bathe and feed my child, and prepare food before leaving and then complete the remaining chores after returning home, just because I chose to work. I’m a female, a daughter in law, a wife, a mother and it is my duty.”

“I kept saying ‘soon, after he goes to school, I can work,’ but that ‘soon’ never comes”

“Sometimes I shout at my child, and immediately regret it. Then I sit and cry after he sleeps.”

"My husband thinks helping once is enough. But parenting is daily and it is not a help!."

“Even a phone call from an employer made me feel torn. I kept thinking, ‘should I be here or there?’ I felt guilty for not being present enough, both at work and home.”

 “I do everything. My husband will help only if I ask 3 times.”

**Participant 3**

“If something went wrong with the child, food habits or any act, I’m blamed. When my child does any good act, the credit goes to my husband. No one said I was a bad mother, but their reactions or indirect comments do.”

"Not one person said I’m not a good mother but everything they do makes me feel it. Earlier women went through domestic violence or verbal abuse, now it's even worse as people outside think I’m happy, I have a family to support or take care of my child and I’m successful as I work but the reality inside the four walls is horrible.”

“Everyone says ‘we’ll help,’ but I end up doing everything. I wanted to switch jobs but felt stuck in a caregiving loop. I’m a JRF candidate and I wished to do a full time Ph. D. I sacrificed my dream and choosing a workplace which runs for half a day is again for the sake of my child.”

“Even if my husband is at work from home, it is my duty to do all the chores and make everything ready for my child before leaving for work. My in-laws don’t allow my husband to help me in childcare just because he is a male”

“Even when I’m sick, I feel guilty resting. Who else will do the work? I was just giving and giving, but nobody noticed.”

“They said the child didn’t eat because I went to work.” "My MIL would say I’m not doing enough just because I gave bottled milk."

**Participant 4**

"I constantly think, ‘Why is only my identity erased in child care?’"

"I was preparing for government exams, I couldn’t even study for two months after delivery."

“Some days I look in the mirror and don’t recognize the person. Not just physically, even mentally I’m not myself.”

**Participant 5**

“After delivery I was left alone to deal with the child. When he cried, I wanted to run away from home.”

“ Even today, my son’s needs are seen as ‘my’ duty. My husband helps, but the mental load is mine. I carry the list. He’ll do it only if I say. Why can’t he think of it himself? ”

“Speech delay, maybe it was because I gave too much screen time to focus on work peacefully. I felt like I compromised his health.”

"No one openly says I’m failing, but there’s always an unspoken judgment."

“When I rejoined work after maternity leave, I was scared they’d think I couldn’t handle work anymore. I kept pushing myself to prove I’m still capable.”

"My career took a hit. I didn’t apply for the promotions I deserved. I once dreamed of being powerful, then one project seemed too much. I was the COO of a company, managing multiple departments.  I slowly gave up my role in office, from COO to managing one project to nothing. I didn’t even realize when I stopped being that person. Now, I’m home all day. I had to quit my job. My plans just disappeared. I always feared: what if I’m failing both my child and my job? I felt I was not doing justice to either. I had to leave work when my son’s speech delay worsened. The guilt of leaving my child was stronger than ambition. I was helpless even though I had my parents and in-laws. They said, "No one can replace a mother and it is my sole responsibility for his condition.”

“I used to be respected at work. Now I doubt myself. I look in the mirror and don’t see someone successful anymore. The longer I stayed home, the harder it became to believe I was worth something outside of motherhood.

“I wonder if I can ever go back to work again. Will I ever be good at my job again? That fear is always there. After leaving work to care for my son’s speech delay, I feel I’ve lost that part of me.”

**Postpartum Identity Disruption (Themes derived)**

Core Theme 1: Disruption of Identity and Selfhood

│

├── Loss of Professional Identity

├── Emotional Flattening and Self-Estrangement

└── Bodily Alienation

Core Theme 2: Emotional Invisibility and Isolation

│

├── Shrinking Social Circles

├── Unseen Emotional Labour

└── Withdrawal and Silence

Core Theme 3: Gendered Labour and Unequal Parenting

│

├── Physical and Cognitive Overload

├── Mental Load and Planning Fatigue

└── Patriarchal Gatekeeping

Core Theme 4: Career Sacrifice and Suppressed Dreams

│

├── Deferred Ambition

├── Professional Disintegration

└── Identity Collapse

Core Theme 5: Cultural Pressure and Maternal Judgment

│

├── Intergenerational Surveillance

├── Moral Policing

└── Internalized Guilt

Core Theme 6: Guilt, Ambivalence, and Emotional Overload

│

├── Structural Guilt

├── Emotional Containment

└── Grief for the Lost Self
